# Valorization of Bilberry (*Vaccinium myrtillus* L.) Pomace by Enzyme-Assisted Extraction: Process Optimization and Comparison with Conventional Solid-Liquid Extraction

**DOI:** 10.3390/antiox10050773

**Published:** 2021-05-13

**Authors:** Michail Syrpas, Egle Valanciene, Ernesta Augustiniene, Naglis Malys

**Affiliations:** 1 Department of Food Science & Technology, Faculty of Chemical Technology, Kaunas University of Technology, Radvilėnų Pl. 19, LT-50254 Kaunas, Lithuania; 2 Bioprocess Research Centre, Faculty of Chemical Technology, Kaunas University of Technology, Radvilėnų Pl. 19, LT-50254 Kaunas, Lithuania; egle.valanciene@ktu.lt (E.V.); ernesta.augustiniene@ktu.lt (E.A.)

**Keywords:** enzyme-assisted extraction, response surface methodology, antioxidants, bilberry pomace, *Vaccinium myrtillus*, food waste

## Abstract

Bilberry (*Vaccinium myrtillus* L.) pomace contains a significant amount of polyphenols and can serve as a basis for food additives, nutraceuticals, and functional foods. Although various techniques can be employed to recover bioactive fractions from berry pomaces, data on enzyme-assisted extraction (EAE) of bilberry pomace are rather scarce. This study aimed to optimize critical EAE parameters using Viscozyme L to obtain a high-yield extract with enhanced antioxidant capacity. Central composite design and response surface methodology evaluating the effect of four independent variables, namely, pH, temperature, extraction time, and enzyme concentration on three responses, were employed to define optimal EAE conditions. Under the optimal conditions (pH: 4.5, temperature 46 °C, 1 h of extraction, and 2 active units (AU) of Viscozyme L/g of pomace), EAE yielded 56.15 g/100 g DW of the water-soluble fraction. Comparison with conventional maceration indicated that EAE, besides the yield, significantly increased the in vitro antioxidant capacity measured by the total phenolic content, ABTS, ORAC, and CUPRAC assays. Moreover, an increase was observed for the measured mono- and disaccharide as well as anthocyanin content. Overall, this study demonstrates the improved efficiency of EAE over conventional solid–liquid extraction to recover fractions with a higher yield and enhanced functional properties in a fast and sustainable manner.

## 1. Introduction

Throughout the world, the processing, consumption, transportation, and storage of agricultural products inevitably leads to the generation of a staggering amount of waste [1]. However, due to the diversity of functionalized molecules that can be recovered from food waste, the latter can constitute an ideal resource for various biobased products or value-added compounds with multiple applications [2]. The idea of food waste valorization through a biorefinery approach, either by recovering bioactive molecules or generating biofuels, aligns perfectly with global goals for sustainable development, which further aims to achieve energy efficiency, environmental protection, and food security [3]. For these reasons, interest in sustainable approaches aiming to valorize these underutilized resources with green technologies remains high.

A considerable amount of literature indicates that berries are a promising functional food, as they are rich in polyphenolic substances, especially anthocyanins [4,5]. *Vaccinium myrtillus* L., commonly known as the European blueberry, huckleberry, or bilberry, is a member of the Ericaceae family. Bilberries contain a plethora of bioactive compounds and nutrients such as anthocyanins, flavonols, flavan-3-ols, stilbenes, procyanidins, tannins, vitamins, and phenolic acids [6,7]. A significant number of polyphenol-rich seeds and skins of berries are typically discarded during bilberry juice production, which results in a relatively lower polyphenol content in the juice. In several cases, cell-wall-degrading enzymes are employed to enhance juice’s polyphenol content and increase the overall yield [8,9]. Nevertheless, the remaining pomace contains a significant amount of polyphenols and can serve as a basis for food additives, nutraceuticals, functional foods, or cosmetics [10,11], or can be used to prepare snacks [12].

Various approaches/techniques have been proposed to efficiently recover antioxidant fractions from bilberry pomace in the last several years. Besides conventional solvent-based extraction techniques [13], emerging technologies such as microwave hydrodiffusion and gravity [14], ultrasound-assisted [10], and pressurized carbon dioxide extraction have been suggested [15] as promising alternatives. Although various techniques can be employed to recover bioactive fractions from bilberry pomaces, data on enzyme-assisted extraction (EAE) are relatively scarce. Recent reviews indicate that among the emerging technologies, EAE is gaining attention as an eco-friendly, sustainable extraction technique for the recovery of antioxidant compounds from agricultural by-products [16,17]. When compared to conventional techniques, EAE offers significant advantages such as higher yield within shorter extraction time, reduced solvent, and energy consumption [18,19,20]. Nevertheless, recovering extracts with higher yields and enhanced properties require a deeper understanding of the applied enzymes’ hydrolytic properties and physicochemical interactions with the feedstock [17]. For these reasons, critical EAE parameters such as pH, temperature, and enzyme concentration that influence bioactive substances’ release require optimization for each specific process [18].

This paper examines the influence and optimization of critical process parameters during EAE with Viscozyme L of bilberry pomace. Viscozyme L is a multienzyme cocktail consisting of pectinases, cellulases, hemicellulases, and arabinases, widely used for the cell-wall breaking of fruit and vegetable products [21]. Toward this end, an experimental approach based on a central composite design (CCD) and response surface methodology (RSM) was employed. Operational parameters such as pH, temperature, extraction time, and enzyme concentration were selected as independent variables, and the EAE process was optimized after evaluation of three dependent variables, namely the total yield, trolox equivalent antioxidant capacity in the ABTS^•+^ assay (TEAC_ABTS_), and total phenolic con-tent (TPC). The use of multiple dependent variables enables optimization, taking into consideration not only the extraction efficiency, in terms of yield, but also qualitative aspects such as the antioxidant capacity of the obtained fractions. Using RSM-defined optimal conditions, EAE was compared to conventional solid–liquid extraction (SLE). Moreover, the in vitro antioxidant capacity and the saccharide and anthocyanin content in EAE fractions were evaluated and directly compared to those obtained using the conventional extraction technique. This study aimed to evaluate and optimize EAE as an efficient strategy to valorize bilberry pomace. This approach could be regarded as a sustainable alternative to obtain higher added-value fractions from agro-industrial residues with potential food, nutraceutical, and pharmaceutical applications.

## 2. Materials and Methods

### 2.1. Bilberry Pomace

Fresh bilberry pomace was obtained, directly after juice pressing, from the local juice producer ‘Obuolių namai’ (Kaunas, Lithuania). The pomace was stored in cooler bags and was subsequently frozen to −18 °C two h after pressing. Frozen pomace was subsequently freeze-dried in an industrial-scale freeze dryer. Dried pomace was then ground by an ultra-centrifugal mill ZM 200 (Retsch, Haan, Germany) using a 0.2 mm hole size sieve. After sieving, dried samples were subjected to supercritical carbon dioxide extraction (SFE-CO_2_) to remove the lipophilic fraction.

SFE-CO_2_ was performed under the optimal extraction pressure and temperature reported for lingonberry (*Vaccinium vitis-idaea* L.) pomace with slight adaptations [22], Briefly, 6.05 kg of bilberry pomace was placed in a 10 L pilot scale extractor (Applied Separation, Allentown, PA) surrounded by a heating jacket that maintained the extraction vessel temperature. A ball float rotameter and a digital mass flow meter were used to measure CO_2_ in SL/min under standard conditions: pressure (P) = 100 kPa, temperature (T) = 20 °C, density (ρ) = 0.0018 g/mL. The static extraction time was 30 min followed by 360 min of total dynamic extraction at 45 MPa and 50 °C with a flow rate of CO_2_ (2 SL/min). Conventional Soxhlet extraction of bilberry pomace was performed in an automated Soxhlet extractor EZ100H (Behr Labor-Technik, Düsseldorf, Germany) using hexane as a solvent. Briely, 10 ± 0.001 g of pomace was extracted with 100 mL of hexane, heated under reflux with a rate of extraction—1 cycle/5 min, for 360 min. The SFE-CO_2_ yielded 6.97 g/100 g DW of lipophilic extract, which represented a 94.4% efficiency as compared to Soxhlet extraction (7.38 ± 0.8 g/100 g DW). Defatted samples were then stored in a dark, well-ventilated space at room temperature until further analysis.

### 2.2. Chemicals and Reagents

6-Hydroxy-2,5,7,8-tetramethylchromane-2-carboxylic acid (TROLOX, 97%), 2,2′-azobis(2-amidinopropane) dihydrochloride (AAPH), Folin–Ciocalteu’s phenol reagent (2 M), gallic acid (99%), d-(+)-glucose (>99%), d-(−)-fructose (>99%), caffeic acid (>98%), and chlorogenic acid (>95%) were obtained from Sigma-Aldrich (Bornem, Belgium); sodium acetate (>99%) from Acros Organics (Geel, Belgium); fluorescein (FL) from Fluka Analytical (Bornem, Belgium); 2,2′-azino-bis(3-ethylbenzothiazoline-6-sulphonic acid) diammonium salt (ABTS^•+^), NaCl, KCl, Na_2_HPO_4_, and K_2_S_2_O_8_ from Merck (Darmstadt, Germany); KH_2_PO_4_ from Jansen Chimica (Beerse, Belgium); and Na_2_CO_3_ (98%, anhydrous) from RPL (Grauwmeen, Belgium). All solvents were of analytical and HPLC-grade grade.

Viscozyme L, originating from *Aspergillus aculeatus*, was purchased from Sigma-Aldrich (Bornem, Belgium). The preparation mainly contains endo-β-1,3- and 1,4-glucanases; the enzymatic activity according to the manufacturer was 128 fungal β-glucanase units per gram (FBG/g). One FBG unit is considered the amount of enzyme preparation needed for barley β-glucan hydrolysis to liberate reducing carbohydrates corresponding to 1 µmol of glucose per min (reaction conditions: pH 5.0, 50 °C, 20 min). For this study, the reported enzyme concentrations are expressed in AU; one AU is considered to be one FBG unit.

### 2.3. Enzyme-Assisted Extraction (EAE) and Solid–Liquid Extraction (SLE)

For enzyme-assisted extractions, 4 g of pomace were weighed in a 50 mL polyethylene centrifugation tube and suspended in 40 mL of a citrate buffer with the selected pH and enzyme concentration value. Along with each sample, three control samples were prepared. For the yield calculations, three blanks were also included, blank A (pomace and buffer), blank B (enzyme and buffer), and blank C (buffer). Prepared mixtures were incubated in a thermostatically controlled shaker (250 rpm) at various temperature and time combinations. Viscozyme L activity was terminated after immersing the centrifugation bottle in boiling water for 10 min, followed by rapid cooling and centrifugation (9000× g, 10 min). The resulting supernatants (water-soluble fractions) and solid residues were collected, freeze-dried, and kept in a freezer (−20 °C) before any further analysis. The amount of water-soluble fraction of bilberry pomace was determined gravimetrically after freeze-drying. SLE of bilberry pomace was performed as described above without the addition of a Viscozyme L preparation.

### 2.4. Central Composite Design (CCD) and Response Surface Methodology (RSM)

Response surface methodology (RSM) using central composite design (CCD) was used to evaluate the effect of the selected independent variables on the EAE extract yields, Trolox equivalent antioxidant capacity in the ABTS^•+^ assay (TEAC_ABTS_), and total phenolic content (TPC), and to identify the optimal conditions for EAE. Design-Expert version 12.0.8.0 (Stat–Ease Inc., Minneapolis, MN, USA) was used to establish the models and analyze the results. All extraction experiments were performed in random order. One-way analysis of the variance (ANOVA) and statistical significance of the model and each variable was determined using the Student’s t-test (*p*-value) at a 5% probability level (*p* < 0.05). The model’s adequacy was determined by evaluating the ‘lack of fit’ coefficient and the Fisher test value (F-value) obtained from ANOVA.

### 2.5. In Vitro Antioxidant Capacity

#### 2.5.1. Total Phenolic Content (TPC)

The obtained bilberry pomace extracts’ total phenolic content was estimated as previously described in the literature [23]. Briefly, 150 μL of sample or blank was mixed with 750 μL of Folin–Ciocalteu’s reagent (1:9, *v*/*v*) followed by 600 μL Na_2_CO_3_ solution (75 g/L). Samples were kept in the dark for two h. The absorbance of optically clear supernatants was measured at 760 nm with a GENESYS 150 UV-Vis Spectrophotometer (Thermo Fisher Scientific, Waltham, MA). The TPC was expressed as gallic acid equivalents (mg GAE/g extract or recalculated per g DW of bilberry pomace residue after SFE-CO2 taking into consideration the extraction yield; mean values ± standard deviation, *n* = 4), employing a dose–response curve (0–80 μg/mL) for gallic acid.

#### 2.5.2. The 2,2′-Azinobis-(3-ethylbenzothiazoline-6-sulfonic acid) (ABTS^•+^) Scavenging Assay

The ABTS^•+^ assay was carried as described elsewhere [24]. Briefly, 50 mL of ABTS^•+^ (2 mmol/L PBS) solution was prepared and mixed with 200 μL K_2_S_2_O_8_ (70 mmol/L). The stock mixture was allowed to stand in the dark at room temperature for 15–16 h prior to dilution. The working solution was then prepared by diluting the stock solution with PBS until a final absorbance of AU 0.700 ± 0.010 at 734 nm was reached. Afterward, 25 μL of extract or blank control sample were mixed with 1500 μL of working radical solution. Then mixtures were vortexed for 15 s and then shaken at 250 rpm in the dark. After two h, samples were centrifuged (1960× *g* for 5 min), and the absorbance of optically clear supernatant was measured. TEAC_ABTS_ was calculated through a dose–response curve for Trolox (0–1500 μmol/L MeOH).

#### 2.5.3. The Oxygen Radical Absorbance Capacity (ORAC) Assay

ORAC of bilberry extracts was measured following previously described procedures [25]. The method utilizes fluorescein as a fluorescent probe. Briefly, 25 μL of the sample or distilled H_2_O used as a blank control sample were mixed with 150 μL of fluorescein solution (14 μmol/L) in a 96-well black opaque microplate. Samples were then preincubated for 15 min at 37 °C. Then, 25 μL of AAPH solution (240 mmol/L) used as a peroxyl radical generator was added. A total of 150 cycles were recorded using a FLUOstar Omega plate reader (BMG Labtech, Offenburg, Germany). Results were expressed as Trolox equivalent antioxidant capacity (TEAC, mg sample) by means of the dose–response curve for Trolox (0–500 μmol/L PBS).

#### 2.5.4. The Cupric Reducing Antioxidant Capacity (CUPRAC) Assay

CUPRAC of bilberry extracts was measured following a previously described procedure with slight modifications [26]. Briefly, 0.6 mL of Cu(II), neocuproine, and NH_4_Ac buffer solutions were added to a test tube. Then, 0.6 mL of extract (or standard) was added to the initial mixture to make the final volume 2.4 mL. The tubes were stoppered, and after 30 min, the absorbance was recorded at 450 nm (760 nm with a GENESYS 150 UV-Vis Spectrophotometer (Thermo Fisher Scientific, Waltham, MA). The CUPRAC antioxidant capacity was expressed as Trolox equivalents (either as mg TE/g extract or per g DW of bilberry pomace residue after SFE-CO_2_, taking into consideration the extraction yield; mean values ± standard deviation, *n* = 4), employing a dose–response curve for Trolox.

### 2.6. Mono- and Disaccharides Analysis by High-Pressure Liquid Chromatography With Refractive Index Detector (HPLC-RI)

Saccharide analysis was performed using a Thermo Scientific Ultimate 3000 HPLC system coupled to a RefractoMax 521 refractive index detector (Thermo Fisher Scientific, Waltham, MA, USA). Saccharide components were separated using two sugar columns in series, SUGAR KS-802 and KS-801 (8.0 mmID × 300 mm each), with ultrapure water as a mobile phase. The columns were operated at 80 °C with an isocratic flow rate of 0.5 mL/min. Samples were run for 60 min, and the injection volume was 10 µL. Chromatograms were recorded and processed using Chromeleon 7 software (Thermo Fisher Scientific, Waltham, MA, USA).

### 2.7. Total Monomeric Anthocyanin Content by the pH Differential Method and HPLC

Total monomeric anthocyanin content was determined spectrophotometrically, using the pH differential method with cyanidin-3-O-glucoside as a standard [27]. Absorbance at 520 and 700 nm was measured with a GENESYS 150 UV-Vis Spectrophotometer (Thermo Fisher Scientific, Waltham, MA, USA). Data were calculated using the molar extinction coefficient for cyanidin-3-glucoside and the equation of Lee et al. [27]. For the HPLC measurements, the analysis was performed using a Thermo Scientific Ultimate 3000 HPLC system coupled to a diode array detector (Thermo Fisher Scientific, Waltham, MA). Chromatographic separation was achieved with a Phenomenex Luna 3μ C18 100Å (250 × 4.60 mm) column (Phenomenex, Aschaffenburg, Germany) equipped with a security guard column, thermostated at 38 °C. Solvent A contained water formic acid (*v*/*v*); solvent B contained acetonitrile with 10% formic acid (*v*/*v*). A constant flow rate of 1 mL/min was kept throughout the analysis with the detection wavelength set at 520 nm. The injection volume for samples or standards was 50 μL. The elution gradients used were as follows: from 0 until 5 min held at 8% B, between 5–25 min 8–32% B; 25–35 raised at 60% B; then kept constant for 2 min, 37–45 min decreased to 8% B kept constant for 5 min. All chromatograms were recorded and analyzed using Chromeleon 7 software. Results were expressed as μg of cyanidin-3-O-glucoside equivalents per g of extract or recalculated per g DW of bilberry pomace residue after SFE-CO_2_ taking into consideration the extraction yield.

### 2.8. Statistical Analysis

Extraction experiments, sugar content, and HPLC analyses were performed in triplicate, whereas antioxidant capacity assessment experiments were at least in quadruplicate. Mean values and standard deviations were calculated using MS Excel 2019 (Microsoft Corp, Albuquerque, NM, USA). Statistical significance was determined using the Student’s t-test (*p*-value) at 5% probability level (*p* < 0.05).

## 3. Results and Discussion

### 3.1. Central Composite Design and Surface Plots of EAE

In this part of the study, we aimed to evaluate and optimize EAE as an efficient strategy to valorize bilberry pomace. This biomass, typically considered as a waste, is rich in antioxidants, and obtained extracts could be of interest to various industries. To enhance extraction efficiency, it is essential to work under optimized conditions. In this sense, several authors have suggested RSM to evaluate and optimize enzymatic hydrolysis factors such as enzyme concentration, temperature, time, and pH [28,29,30]. Herein, in order to select optimal extraction conditions, the effects of four independent variables, namely temperature (°C), pH, time (hours), and enzyme concentration (AU/g), on the EAE extract yield (g/100 g DW), TPC, and TEAC_ABTS_ were determined employing RSM and CCD. The range of the experimental conditions evaluated for the independent variables was based on previous publications [29,30,31].

**Figure 1:**
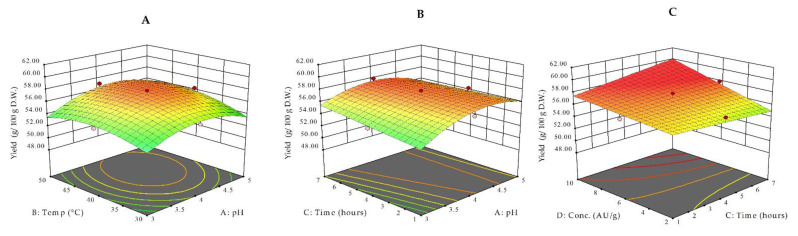
3D response surface plots for extraction yield as a function of temperature and pH (at a constant time of 4 h and at a concentration of 6 AU/g) (**A**), time and pH (at a constant temperature of 40 °C and at an enzyme concentration of 6 AU/g) (**B**), and enzyme concentration and time (at a constant temperature of 40 °C and at a pH of 4) (**C**).

The main and interactive effects of the studied independent variables (pH, temperature, time, and enzyme concentration) on the three dependent variables (RFI, II, and III) were analyzed by response surface methodology. 3D plots visualize the interaction of two independent variables and their influence on the selected response. For example, Figure 1A shows the effect of pH and temperature at a fixed extraction time (4 h) and enzyme concentration (6 AU/g).

From the interaction contour plots, it is evident that the yield of the water-soluble fraction of bilberry pomace treated with Viscozyme L is strongly influenced by all independent variables (Figure 1A–C). Figure 1A shows that the pH value in the region between 4–4.5 and temperatures between 40–45 °C have a positive effect on the yield. The influence of time and pH at a fixed temperature (40 °C) and enzyme concentration (6 AU/g) is depicted in Figure 1B, whereas Figure 1C shows the response surface plots as a function of enzyme concentration and time. As it could be expected, there is a strong positive correlation between yield and higher applied extraction times and enzyme concentrations (Figure 1C). Under various experimental conditions, the yield ranged between 49.43–58.04 g/100 g DW (Table 1). The three highest yield values were all observed under treatments performed at a pH of 4 and 40 °C, with different combinations of extraction time and enzyme concentration (Table 1).

**Figure 2:**
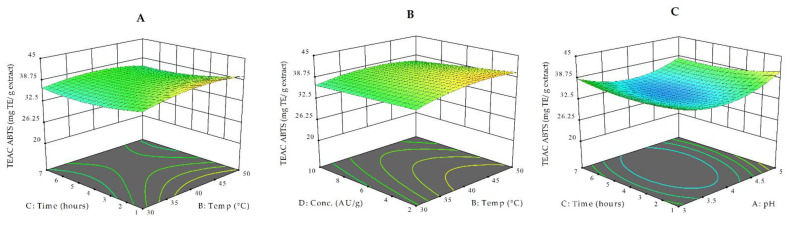
3D response surface plots for TEAC_ABTS_ as a function of temperature and time (at a constant enzyme concentration of 6 AU/g and at a pH of 4.5) (**A**), temperature and enzyme concentration (at a constant time of 2 h and at a pH of 4.5) (**B**), and extraction time and pH (at a constant temperature of 40 °C and at an enzyme concentration of 6 AU/g) (**C**)**.**

Figure 2A–C present the response surface plots for TEAC_ABTS_ values, as measured by the ABTS^•+^ radical scavenging capacity assay. TEAC_ABTS_ values, expressed as mg of TE/g of extract, varied under different enzymatic treatments from 32.56 to 42.70 (Table 1). As seen in Figure 2A,B, the tested range of temperature (30–50 °C) did not significantly influence the different treatments’ TEAC_ABTS_ values. In fact, ANOVA results show that the temperature was not a statistically significant model term (Supplementary Material, Table S1). ABTS^•+^ scavenging activity of the obtained extracts showed the highest activity after 1 h of enzyme hydrolysis at a pH of 5 and temperature of 50 C, followed by a progressive decrease up to 7 h of extraction [29]. A similar observation was reported by Kapasakalidis et al. after Celluclast treatments of black currant pomace. Generally, the TEAC_ABTS_ values of EAE extracts were higher with increasing time over the studied pH scale (pH × time; *p* = 0.0039). Enzyme concentration, pH, and time were significant in determining the radical scavenging activity of the water-soluble fractions of bilberry pomace after EAE (Supplementary Materials, Table S1).

**Figure 3:**
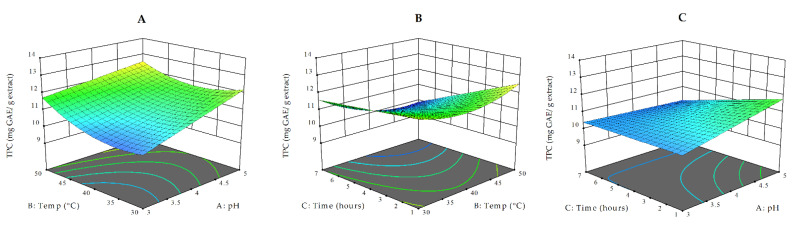
3D response surface plots for TPC as a function of temperature and pH (at a constant time of 4 h and at an enzyme concentration of 6 AU/g) (**A**), temperature and time (at a constant pH of 4 and at an enzyme concentration of 6 AU/g) (**B**), and extraction time and pH (at a constant temperature of 40 °C and at an enzyme concentration of pH of 6 AU/g) (**C**).

Figure 3 presents the 3D response surface plots for TPC as a function of various independent variables. The TPC was measured by the Folin–Ciocalteu assay, and the results were expressed as mg of GAE/g of extract. Similar to the TEAC_ABTS,_ the highest TPC value (13.26 mg GAE/g extract) was obtained under treatment at 50 °C and a pH of 5 (Table 1). The lowest TPC values were observed in the treatments performed at a pH of 4 and temperature of 40 °C (runs 2, 3, and 16, Table 1). The higher (10 AU/g pomace) and lower (2 AU/g pomace) enzyme concentration treatments resulted in quite similar values of TPC over an increasing period of extraction time, as also suggested by the lack of significance (*p* = 0.13) in the interaction of these terms (Supplementary Materials, Table S1).

### 3.2. Model Validation and Simultaneous Response Optimization by the Desirability Function

The analysis of variance (ANOVA) was used to determine the adequacy of the developed models. ANOVA indicated that all the proposed models were significant (*p* < 0.0001) with F-values of 46.12, 13.95, and 13.81 for RF I, II, and III, respectively (Supplementary Materials, Table S1). The models’ adequacy, also depicted by the lack of fit F-values, showed that this parameter is not significant compared to the pure error (*p* > 0.05), which is the desired characteristic (Supplementary Materials, Table S1). The determination coefficients (R^2^) are presented in Table S2 Supplementary Materials. However, since a sizeable R^2^ value does not always indicate a sound regression model, attention should also be given to the adjusted and predicted R^2^ values [32]. As presented in Table S2, the difference between adjusted and predicted R^2^ for all RFs was less than 0.2, indicating a reasonable agreement (Supplementary Materials, Table S2). Table S3 in the Supplementary Material presents the regression coefficients of the intercept, linear, interaction, and quadratic terms of all models (Supplementary Materials, Table S3). The ANOVA results indicate that all linear parameters were significant (*p* < 0.05) for RF I and II, whereas for RF III, temperature and enzyme concentration were not significant factors (*p* > 0.05) (Supplementary Materials, Table S1). After removal of non-significant parameters (*p* > 0.05), the final reduced predictive equations describing the polynomial models can be summarized in Equations (1)–(3) presented below:

RFI (YIELD) = −3.85902 + 16.1291 ∗ A + 1.01033 ∗ B + 0.00375671 ∗ C + 1.43413 ∗ D + 0.0410812 ∗ AB + -0.201546 ∗ AC + −0.104878 ∗ AD + 0.0119263 ∗ BC + −0.0227822 ∗ BD + 0.075601 ∗ CD + −1.95419 ∗ A^2 + −0.0127244 ∗ B^2(1)

RFII (TEACABTS) = 54.1706 + −25.6618 ∗ A + 1.37555 ∗ B + 0.384843 ∗ C + 0.673317 ∗ D + −0.24819 ∗ AC + −0.0953141 ∗ AD + −0.017201 ∗ BC + −0.0117961 ∗ BD + 3.47321 ∗ A^2 + −0.0149554 ∗ B^2 + 0.140418 ∗ C^2(2)

RFIII (TPC) = 12.1778 + 1.87305 ∗ A + −0.314582 ∗ B + 1.018 ∗ C + −0.375587 ∗ D + −0.0265587 ∗ AB + −0.13485 ∗ AC + −0.0171875 ∗ BC + 0.0122125 ∗ CD + 0.00602362 ∗ B^2 + 0.0277414 ∗ D^2(3)

where A = pH (3–5), B = temperature (30–50 °C), C = time (1–7 h) and D = enzyme concentration (2–10 AU/g)

Overall, it can be concluded that ANOVA results verified the adequacy of the suggested three models for yield, TEAC_ABTS_, and TPC, to forecast the relationship between the studied extraction process parameters and the various responses within the selected experimental domain. Considering all observed responses, EAE was optimized based on the numerical optimization and the desirability function within the selected range of variables. In Design-Expert software, for several responses and factors, all goals were combined into one desirability function, and the numerical optimization enabled to predict a point that maximizes the overall desirability depending on the chosen restraints. For this study, two of the experiment’s independent variables, namely pH and temperature, were set as “in range”, whereas enzyme concentration and extraction time were set as “minimize”. The latter two parameters were selected to reduce as much as possible the overall production cost, since especially the enzyme cost is a limiting factor for commercial applications of EAE [18]. Based on these criteria, the application of the desirability function enabled simultaneous optimization of all the responses. The suggested optimal extraction conditions were pH = 4.5, a temperature of 46 °C, and extraction time of 1 h, with an enzyme concentration of 2 AU/g of pomace. The overall desirability, under these conditions, was 0.933. After optimizing the EAE process, we carried out the fitting models’ authenticity for all responses. Experimental validation, employing three replicates, confirmed the model’s ability to predict the studied responses, yield, TEAC_ABTS_, and TPC (Supplementary Material, Table S4). Under the optimal conditions, an extract with a yield of 56.15 g/100 g DW, 12.15 mg GAE/g DW, and 37.82 mmol TE/g DW was obtained, which was in agreement with the model prediction (Table 2 and Table S4).

### 3.3. Comparison of Enzyme-Assisted Extraction With Solid–Liquid Extraction and In Vitro Antioxidant Capacity

In this part of the research, EAE was compared to conventional maceration under the previously defined by RSM optimal extraction conditions. SLE was performed at a pH of 4.5 and temperature 46 °C for 1 h, but excluding Viscozyme L to further evaluate the enzymatic treatment’s influence. Significantly, EAE yielded 56.15 g of water-soluble material per 100 g DW under these conditions. This is an approximately 30% increase compared to the 43.10 g/100 g DW yielded by the conventional SLE technique. In this study, a significant increase in yield is observed within a relatively short extraction time (60 min) at the lowest tested enzyme concentration (2 AU/g of pomace). It has to be mentioned that EAE, under various treatments, also led to a yield increase that ranged from 20–38% as compared to control samples without the enzyme addition (data not shown). Kitrytė et al., using similar enzymatic treatments, reported an increase of 24–80% and 44–113% for seabuckthorn [31] and chokeberry pomace’s water-soluble fraction yield, respectively [30]. Overall, these results are consistent with previous reports showing that EAE significantly enhances the yield of recoverable fractions at short extraction times [18,19,20].

The yield increase observed in EAE is in multiple cases accompanied by an enhanced recovery of polyphenolic substances and an increase of the antioxidant capacity [33,34]. Toward this, we evaluated the in vitro antioxidant capacity of bilberry extracts by four widely utilized assays (Table 2). Besides the two assays that were part of the experimental design (TPC and TEAC_ABTS_), the extracts’ in vitro antioxidant capacity was also measured using the ORAC and CUPRAC assays. Although these assays, similar to other in vitro assays, come with certain limitations, they offer specific advantages. For example, ORAC utilizes a hydrogen atom transfer radical quenching mechanism of peroxyl radicals, which are considered better models of antioxidant reactions with reactive oxygen species both in foods and in vivo [35]. CUPRAC, on the other hand, offers distinct advantages over other electron transfer-based protocols such as TPC, notably the selective oxidation of antioxidant compounds without reacting with sugars and organic acids commonly present in food products or their extracts [26]. As shown by the results presented in Table 2, EAE increased the overall antioxidant capacity significantly. When recalculated per g of DW of pomace, the % increase was approximately 30% for the TEAC_ABTS_, TPC, and CUPRAC assays and approximately 10% for the ORAC assay, indicating an enhanced recovery of antioxidant substances (Table 2).

The plant cell wall structure consists of complex structural polysaccharides that confer the cell’s stability and resistance to the intracellular components’ extraction [33]. Phenolic substances are known to be present in two forms, free and bound. The free form can be easily and quickly recovered even by simple extraction techniques such as maceration. When hydrolytic enzymes, including the one used in this study, are applied, the enzyme’s cell wall degrading activity can lead to the release of chemically bound and physically entrapped phenolics present in food matrixes and intact cells, thus leading to a substantial increase in the observed antioxidant capacity [36,37].

### 3.4. Comparison of Mono- and Disaccharide Content

To evaluate the influence of Viscozyme L on the release and content of simple carbohydrates, the mono- and disaccharide concentrations in the EAE and SLE extracts, obtained under optimal conditions, were measured by HPLC-RI. Table 3 presents the glucose, sucrose, and fructose content of the analyzed samples. As it could be expected, the enzyme’s hydrolytic activity is reflected in the substantial increase of the saccharide content, especially glucose (Table 3). Cell-wall polysaccharides of bilberry pomace contain pectin, hemicellulose (mostly xylan), and cellulose, all of which could serve as Visczoyme’s substrates [38]. To a large extent, the substantial four-fold increase of glucose content in EAE is probably related to the cellulase activity, hydrolyzing the glucose β-1,4 linked polymer. In this study, the monosaccharide content after EAE was 109.55 and 121.87 mg/g of extract for glucose and fructose, respectively. This result is in close agreement with a previous study with chokeberry pomace, where the authors reported that under various hydrolytic enzyme treatments, the glucose content ranged from 104.8–186.1 mg/g extract, whereas the fructose content varied from 91.7 to 122.6 mg/g [30]. Under the optimal extraction conditions, the influence of the hydrolytic activity of Viscozyme L. is also depicted in the total mono and disaccharide content reported in this study (Table 3). Specifically, SLE amounted to 74.48 mg/g DW, whereas for EAE, the total content was 132.45 mg/g DW. Although various factors can influence the soluble and insoluble fiber composition of bilberry press cake [39], these results agree with a previous report on bilberry pomace valorization. Zhou et al. reported that the total mono- and disaccharide content of bilberry press cake was around 7% for Soxhlet extraction with water [40], which is in agreement with the SLE value (74.48 mg/g DW) of this study. Moreover, the authors reported a total yield of 24% after 30 min of microwave hydrolysis treatment, which is higher than the ≈13% of the enzymatic hydrolysis treatment presented here [40]. Further studies focusing on the influence of the enzymatic treatment on the soluble and insoluble dietary fiber content, degree of polymerization, and individual component content could provide useful insight for the potential application of the obtained EAE extract as a source of dietary fiber.

### 3.5. Comparison of Total Anthocyanin Content

Total anthocyanin content, expressed as cyanidin-3-*O*-glucoside equivalents, of the obtained bilberry extracts by EAE and SLE was assessed using the pH differential method and HPLC-UV (Table 4). Although the spectrophotometric method underestimates the total content by 2–5 times, depending on the matrix, these methods show a high correlation [41]. In this study, the total anthocyanin content as measured by the HPLC method was approximately four times higher than the spectrophotometric observation (Table 4). Nevertheless, both techniques are widely used by researchers and laboratories globally to quantify anthocyanins in a sample or assess the quality of fresh and processed fruits [41]. Especially for laboratories that do not have the HPLC analysis capability, the colorimetric technique offers a simple and economical method for anthocyanin determination [27]. For bilberry and bilberry pomace, similarly to many other fruits, anthocyanin and other secondary metabolite contents largely depend on multiple environmental factors, especially sunlight exposure, altitude, habitat type, and soil carbon content [42].

Moreover, the selected extraction solvent and technique play a significant role in the recovery efficiency. For example, Ravi et al. reported a three-fold increase when bilberry pomace samples were extracted with pure ethanol instead of water [14]. In this study, the HPLC method’s total anthocyanin content was 3.70 and 3.19 mg/g of extract for SLE and EAE, respectively, which is in close agreement with Ravi et al.’s report [14]. When the total anthocyanin content is recalculated per g of DW, the EAE showed a higher content for both techniques (spectrophotometric: 0.4 mg/g DW, HPLC: 1.8 mg/g DW). Although direct comparison is not always possible, these values are in line with previous studies. Kerbstadt et al. reported a total anthocyanin content of 13.67 mg/g DW in the extract that was recovered by supercritical carbon dioxide with ethanol as a co-solvent from freeze-dried bilberries with a 6% moisture content [43]. Moreover, the total monomeric anthocyanin content of eight different-colored and non-pigmented bilberry samples from Finland was found in a range between 2.06–8.67 mg/ g DW by the Colak et al. study [44]. Similarly, Varo et al. showed that the anthocyanin content of bilberry press residues was ≈6.5 mg/g DW after 60 min of maceration and was reduced (≈3.5–5.5 mg/g DW) with different ultrasound treatments [45]. From a qualitative perspective, the two extracts did not reveal any significant differences, as depicted in the overlaid chromatograms (Supplementary Materials, Figure S1). In both cases, the major peaks are related to compounds eluting at a retention time of 17.4 min (λmax: 523 nm), 17.9 min (λmax: 526 nm), and 18.6 min (λmax: 527 nm) (Supplementary Materials, Figure S1). Qualitative determination of anthocyanins relying on UV detection without synthetic standards or reliable mass spectrometry data is rather difficult. Previous studies have shown that the anthocyanin composition of bilberries is characterized by glycosylated, mainly the galactose, glucose, and arabinose derivatives of delphinidin, cyanidin, petunidin, malvidin, and peonidin [46,47]. Nevertheless, our study demonstrates that EAE can also be utilized to recover anthocyanins. Furthermore, the bilberry pomace can be used as a potential source of anthocyanins with multiple applications.

## 4. Conclusions

This study further confirms the great potential of bilberry pomace, among other agro-industrial by-products, as an economical source of bioactive agents with multiple prospective applications. EAE proved to be a good alternative for the recovery of antioxidant fractions from this underutilized by-product. After CCD and RSM, the optimal extraction conditions for EAE bilberry pomace were pH of 4.5, extraction temperature of 46 °C, enzyme concentration of 2 AU/g of pomace, and 1 h of extraction time. Under these conditions, a water-soluble fraction with high yield and antioxidant capacity was obtained. Besides a substantial increase of the water-soluble fraction yield, EAE showed an enhanced recovery of saccharide content and a significant increase of the in vitro antioxidant capacity and anthocyanin content compared to conventional maceration. Overall, this study demonstrates the efficiency of EAE in recovering fractions with high yield and enhanced functional properties in a fast and sustainable manner. Future research investigating the influence of the enzymatic treatment on the soluble and insoluble dietary fiber content, as well as the recovery and fate of specific phenolic substances after hydrolysis, can further advance the use and application range of enzyme-assisted technologies in the agro-industrial waste valorization.

## Figures and Tables

**Table 1:** Central composite design matrix for the enzyme-assisted extraction bilberry pomace and values of observed response factors RFI (Yield, g/100 g of residue after SFE-CO_2_), RFII (TEAC_ABTS_, mg TE/g of extract), and RFIII (TPC, mg GAE/g of extract).

						RF I	RF II	RF III
		pH	Temperature	Time	E/L	Yield	TEAC_ABTS_	TPC
Run	Space Type		° C	h	AU/g	g/100 g DW	mg TE/g Extract	mg GAE/g Extract
1	Factorial	3	50	1	10	52.78	36.73	12.27
2	Axial	4	40	7	6	57.91	35.96	10.21
3	Center	4	40	4	6	57.13	35.65	9.91
4	Factorial	3	30	7	2	49.94	37.06	11.79
5	Factorial	3	50	7	2	52.44	36.99	10.85
6	Factorial	3	30	1	2	50.57	36.22	10.69
7	Factorial	5	50	7	2	54.64	38.5	10.35
8	Axial	4	40	1	6	56	35.51	11.71
9	Factorial	3	50	1	2	51.58	38.42	12.22
10	Factorial	3	50	7	10	57.09	36.54	11.44
11	Axial	4	50	4	6	57.05	32.57	10.98
12	Axial	4	40	4	2	56.26	33.63	11.32
13	Factorial	5	30	7	2	49.43	36.93	11.66
14	Factorial	5	50	7	10	56.67	34.95	10.54
15	Axial	3	40	4	6	54.13	37.95	10.58
16	Center	4	40	4	6	56.95	33.24	10.28
17	Factorial	3	30	1	10	54.46	35.08	10.55
18	Factorial	5	30	7	10	56.4	36.22	12.23
19	Center	4	40	4	6	57.03	34.3	10.4
20	Factorial	5	30	1	10	55.6	37.95	12.65
21	Axial	5	40	4	6	56.29	37.95	11.54
22	Axial	4	30	4	6	54.73	33.39	11.67
23	Factorial	3	30	7	10	57.27	36.93	11.98
24	Axial	4	40	4	10	58.04	33.6	11.01
25	Center	4	40	4	6	57.86	33.42	10.71
26	Factorial	5	50	1	2	56.28	42.7	13.26
27	Factorial	5	30	1	2	52.77	38.65	12.55
28	Factorial	5	50	1	10	54.83	38.14	12.46

TEAC: Trolox equivalent antioxidant capacity, E/L: enzyme concentration; RF: response factor; SFE-CO_2_: supercritical carbon dioxide extraction; SS: the sum of square; df: the degree of freedom; MS: mean square; F: Fisher value; GAE: gallic acid equivalents; TPC: total phenolic content.

**Table 2:** Comparison of the yield and in vitro antioxidant capacity of the water-soluble fractions of bilberry pomace obtained using SLE and EAE.

Antioxidant Assay		SLE	EAE
	Yield (g/100 g DW)	43.1 ± 0.6 ^a^	56.1 ± 0.7 ^b^
TEAC_ABTS_	mg TE/g extract	36.7 ± 0.5 ^a^	37.8 ± 1.2 ^a^
	mg TE/g DW	15.8 ± 0.2 ^a^	21.2 ± 0.6 ^b^
TPC	mg GAE/g extract	12.4 ± 0.8 ^a^	12.1 ± 0.2 ^a^
	mg GAE/g DW	5.3 ± 0.3 ^a^	6.8 ± 0.1 ^b^
ORAC	mg TE/g extract	50.2 ± 0.4 ^a^	42.3 ± 0.4 ^b^
	mg TE/g DW	21.6 ± 0.2 ^a^	23.78 ± 0.2 ^b^
CUPRAC	mg TE/g extract	20.4 ± 0.4 ^a^	19.49 ± 0.2 ^a^
	mg TE/g DW	8.8 ± 0.2 ^a^	10.94 ± 0.1 ^b^

Values are reported as mean ± St. Dev (*n* = 4) and are expressed as mg of TE or GAE per g of extract and recalculated per g DW of bilberry pomace residue after SFE-CO_2_ taking into consideration the extraction yield. TEAC: Trolox equivalent antioxidant capacity, GAE: gallic acid equivalents, TE: Trolox equivalents. Different superscript letters within the same line indicate significant differences (*p* < 0.05).

**Table 3:** Mono- and disaccharide content of the water-soluble fractions of bilberry pomace obtained using SLE and EAE.

	SLE	EAE
**Sucrose (mg/g extract)**	7.1 ± 0.40 ^a^	4.5 ± 0.3 ^b^
**Sucrose (mg/g DW)**	3.1 ± 0.17 ^a^	2.5 ± 0.2 ^b^
**Glucose (mg/g extract)**	33.7 ± 1.40 ^a^	109.5 ± 1.4 ^b^
**Glucose (mg/g DW)**	14.5 ± 0.60 ^a^	61.5 ± 0.8 ^b^
**Fructose (mg/g extract)**	132.1 ± 6.06 ^a^	121.9 ± 4.7 ^b^
**Fructose (mg/g DW)**	56.9 ± 2.61 ^a^	64.8 ± 2.7 ^b^
**Total (mg/g extract)**	172.8	235.9
**Total (mg/g DW)**	74.5	132.4

Values are reported as mean ± St. Dev (*n* = 3) and are expressed as mg per g of extract and recalculated per g DW of bilberry pomace residue after SFE-CO_2_, taking into consideration the extraction yield. Different superscript letters within the same line indicate significant differences (*p* < 0.05).

**Table 4:** Comparison of total monomeric anthocyanin content of water-soluble fractions of bilberry pomace as measured spectrophotometrically by the pH differential method and HPLC-DAD.

Method	SLE	EAE
Spectrophotometer (μg cyan-glu/g)	875.0 ± 30.5 ^a^	748.1 ± 14.6 ^b^
Spectrophotometer (μg cyan-glu/g DW)	377.1 ± 13.1 ^a^	420.1 ± 8.2 ^b^
HPLC (μg cyan-glu/g)	3713.7 ± 95.2 ^a^	3194.0 ± 123.6 ^b^
HPLC (μg cyan-glu/g DW)	1600.6 ± 41.0 ^a^	1793.9 ± 69.42 ^b^

Values are reported as mean ± St. Dev (*n* = 3) of μg of cyanidin-3-o-glucoside equivalents per g of extract and recalculated per g DW of bilberry pomace residue after SFE-CO2 taking into consideration the extraction yield. Different superscript letters within the same line indicate significant differences (*p* < 0.05).

## Data Availability

Not applicable.

## Supplementary Materials

The following are available online at https://www.mdpi.com/article/10.3390/antiox10050773/s1, **Table S1**. Analysis of variance of the regression parameters for EAE response surface quadratic models of bilberry pomace for the response factors extract yield (g/100 g of residue after SFE-CO_2_), TEAC_ABTS_ and total phenolic content (mg GAE/g of residue after SFE-CO_2_). **Table S2** Fit statistics for the proposed models. **Table S3** Coefficients in terms of coded factors for the proposed models. **Table S4**. Validation of predictive models. **Figure S1**. HPLC chromatograms at 520 nm of SLE and EAE extracts under optimal conditions. (A) SLE extract (B) EAE extract and (C) overlaid chromatograms.

## Abbreviations

ABTS^•+^: 2:2′-azinobis-(3-ethylbenzothiazoline-6-sulfonic acid); ANOVA: analysis of variance AU: active units; cyan-glu: cyanidin-3-o-glucoside; CCD: central composite design; CUPRAC: cupric reducing antioxidant capacity; EAE: enzyme-assisted extraction; FBG: fungal β-glucanase; GAE: gallic acid equivalents; h: hours; HPLC: high pressure liquid chromatography; ORAC: oxygen radical absorbance capacity; RI: refractive index; SFE-CO_2_: supercritical carbon dioxide extraction; SLE: solid liquid extraction; TE: Trolox equivalents; TEAC: Trolox equivalent antioxidant capacity; TROLOX: 6-Hydroxy-2,5,7,8-tetramethylchromane-2-carboxylic acid; TPC: total phenolic content.
