# Supplementary material for: Valorization of Bilberry (Vaccinium myrtillus L.) Pomace by Enzyme-Assisted Extraction: Process Optimization and Comparison with Conventional Solid-Liquid Extraction
Source: Antioxidants (Basel). 2021 May 13;10(5):773. doi: 10.3390/antiox10050773 (PMC8152979; doi:10.3390/antiox10050773)
Supplement: Supplementary file 1 [file antioxidants-10-00773-s001.zip › antioxidants-1205478-supplementary.pdf]

Supplementary Materials

Valorization of bilberry (*Vaccinium myrtillus* L.) pomace by enzyme-assisted extraction: process optimization and comparison with conventional solid-liquid extraction

Michail Syrpas [1,2]*, Egle Valanciene [2], Ernesta Augustiniene [2] and Naglis Malys [2*]

[1] Department of Food Science & Technology, Faculty of Chemical Technology, Kaunas University of Technology, Radvilėnų pl. 19, LT-50254 Kaunas, Lithuania; michail.syrpas@ktu.lt (M.S.)
[2] Bioprocess Research Centre, Faculty of Chemical Technology, Kaunas University of Technology, Radvilėnų pl. 19, Kaunas LT-50254, Lithuania; egle.valanciene@ktu.lt (E.V.); ernesta.augustiniene@ktu.lt (E.A); naglis.malys@ktu.lt (N.M)
* Correspondence: michail.syrpas@ktu.lt; naglis.malys@ktu.lt; Tel.: (+37062341846)

Supplementary Tables

Table S1. Analysis of variance of the regression parameters for EAE response surface quadratic model of bilberry pomace for the response factors extract yield (g/100 g of residue after SFE-CO_2), $TEAC_{ABTS}$ and total phenolic content (mg GAE/g of residue after SFE-CO2)

Source	Sum of Squares	df	Mean Square	F-value	*p*-value	
**RF I: EAE extract yield (g/100 g of Residue after SFE-CO2)**						
Model	119.92	11	10.9	13.95	< 0.0001	*
A-Ph	5.63	1	5.63	7.2	0.0163	*
B-Temperature (T, °C)	2.81	1	2.81	3.6	0.0759	*
C-Time (τ, hours)	4.83	1	4.83	6.18	0.0244	*
D-Enzyme load (AU/g)	9.31	1	9.31	11.91	0.0033	*
AC	8.87	1	8.87	11.35	0.0039	*
AD	2.33	1	2.33	2.98	0.1038	**
BC	4.26	1	4.26	5.45	0.0329	*
BD	3.56	1	3.56	4.56	0.0486	*
A^2	33.97	1	33.97	43.46	< 0.0001	*
B^2	6.3	1	6.3	8.06	0.0119	*
C^2	4.5	1	4.5	5.75	0.029	*
Residual	12.5	16	0.7815			
Lack of Fit	8.85	13	0.6811	0.5599	0.8006	**
Pure Error	3.65	3	1.22			
Cor Total	132.43	27				
**RF II: $TEAC_{ABTS}$ (mg TE/ g Extract)**						
Model	119.92	11	10.9	13.95	< 0.0001	*
A-Ph	5.63	1	5.63	7.2	0.0163	*
B-Temperature (T, °C)	2.81	1	2.81	3.6	0.0759	*
C-Time (τ, hours)	4.83	1	4.83	6.18	0.0244	*
D-Enzyme load (AU/g)	9.31	1	9.31	11.91	0.0033	*
AC	8.87	1	8.87	11.35	0.0039	*
AD	2.33	1	2.33	2.98	0.1038	**
BC	4.26	1	4.26	5.45	0.0329	*
BD	3.56	1	3.56	4.56	0.0486	*

A^2	33.97	1	33.97	43.46	< 0.0001	*
B^2	6.3	1	6.3	8.06	0.0119	*
C^2	4.5	1	4.5	5.75	0.029	*
Residual	12.5	16	0.7815			
Lack of Fit	8.85	13	0.6811	0.5599	0.8006	**
Pure Error	3.65	3	1.22			
Cor Total	132.43	27				
**RF III: TPC (mg GAE/ g extract)**						
Model	18.73	10	1.87	13.81	< 0.0001	*
A-Ph	1.32	1	1.32	9.76	0.0062	*
B-Temperature (T, °C)	0.1061	1	0.1061	0.7818	0.3889	**
C-Time (τ, hours)	2.98	1	2.98	21.96	0.0002	*
D-Enzyme load (AU/g)	0.0109	1	0.0109	0.0805	0.78	**
AB	1.13	1	1.13	8.32	0.0103	*
AC	2.62	1	2.62	19.3	0.0004	*
BC	4.25	1	4.25	31.35	< 0.0001	*
CD	0.3436	1	0.3436	2.53	0.13	**
B^2	1.23	1	1.23	9.03	0.008	*
D^2	0.6655	1	0.6655	4.9	0.0407	*

**: significant; **: not significant; EAE: enzyme-assisted extraction; RF: response factor; SFE-CO2: supercritical carbon dioxide extraction; SS: the sum of square; df: the degree of freedom; MS: mean square; F: Fisher value; GAE: gallic acid equivalents; TPC: total phenolic content.*

Table S2. Fit statistics for the proposed models

	**RF I**	**RF II**	**RF III**
St. Dev.	0.54	0.88	0.37
Mean	55.08	36.26	11.35
C.V. (%)	0.99	2.44	3.25
R^2	0.9736	0.9056	0.8904
Adjusted R^2	0.9525	0.8407	0.8259
Predicted R^2	0.9030	0.7331	0.7658
Adeq Precision	24.39	16.31	12.76

St. Dev: standard deviation, C.V.: coefficient of variation, RF I: EAE extract yield (g/100 g of residue after SFE-CO2), RF II: TEACABTS (mg TE/ g extract), RF III: TPC (mg GAE/ g extract)

Table S3 Coefficients in terms of coded factors for the proposed models

**Factor**	**CE**	**df**	**SE**	**95% CI low**	**95% CI High**	**VIF**
RF I: EAE extract yield (g/100 g of residue after SFE-CO2)						
Intercept	57.15	1	0.1821	56.76	57.54	
A-ph	0.7034	1	0.1283	0.4298	0.9769	1
B-Temperature	0.6771	1	0.1283	0.4036	0.9507	1
C-Time	0.3847	1	0.1283	0.1111	0.6582	1
D-Enzyme load	1.62	1	0.1283	1.35	1.9	1
AB	0.4108	1	0.1361	0.1207	0.7009	1
AC	-0.6046	1	0.1361	-0.8948	-0.3145	1
AD	-0.4195	1	0.1361	-0.7096	-0.1294	1
BC	0.3578	1	0.1361	0.0677	0.6479	1
BD	-0.9113	1	0.1361	-1.2	-0.6212	1
CD	0.9072	1	0.1361	0.6171	1.2	1
A^2	-1.95	1	0.2963	-2.59	-1.32	1.9

B^2	-1.27	1	0.2963	-1.9	-0.641	1.9
RF II: TEAC$_{ABTS}$ (mg TE/ g extract)						
Intercept	34.17	1	0.3022	33.53	34.81	
A-ph	0.5593	1	0.2084	0.1176	1	1
B-Temperature	0.3954	1	0.2084	-0.0463	0.8371	1
C-Time	-0.5178	1	0.2084	-0.9596	-0.0761	1
D-Enzyme load	-0.7191	1	0.2084	-1.16	-0.2774	1
AC	-0.7446	1	0.221	-1.21	-0.276	1
AD	-0.3813	1	0.221	-0.8498	0.0873	1
BC	-0.516	1	0.221	-0.9846	-0.0475	1
BD	-0.4718	1	0.221	-0.9404	-0.0033	1
A^2	3.47	1	0.5268	2.36	4.59	2.28
B^2	-1.5	1	0.5268	-2.61	-0.3787	2.28
C^2	1.26	1	0.5268	0.1469	2.38	2.28
RF III: TPC (mg GAE/ g extract)						
Intercept	10.68	1	0.1232	10.42	10.94	
A-ph	0.2713	1	0.0868	0.0881	0.4545	1
B-Temperature	-0.0768	1	0.0868	-0.26	0.1064	1
C-Time	-0.4069	1	0.0868	-0.5901	-0.2237	1
D-Enzyme load	0.0246	1	0.0868	-0.1585	0.2078	1
AB	-0.2656	1	0.0921	-0.4599	-0.0713	1
AC	-0.4045	1	0.0921	-0.5988	-0.2103	1
BC	-0.5156	1	0.0921	-0.7099	-0.3213	1
CD	0.1466	1	0.0921	-0.0477	0.3408	1
B^2	0.6024	1	0.2004	0.1795	1.03	1.9
D^2	0.4439	1	0.2004	0.021	0.8667	1.9

CE: coefficient estimate, df: degrees of freedom; SE: standard error, CI: confidence interval, VIF: Variance inflation factor

Table S4. Validation of predictive models

RF	Predicted Mean	Predicted Median	Std Dev	n	SE Pred	95% PI Low	Data Mean	95% PI high
RFI: Yield	56.83	56.83	0.54	3.00	0.45	55.87	56.15	57.79
RFII: TEAC $_{ABTS}$	38.68	38.68	0.88	3.00	0.77	37.05	37.82	40.31
RFIII: TPC	12.39	12.39	0.37	3.00	0.31	11.73	12.15	13.05

RF I: EAE extract yield (g/100 g of residue after SFE-CO2), RF II: TEACABTS (mg TE/ g extract), RF III: TPC (mg GAE/ g extract)

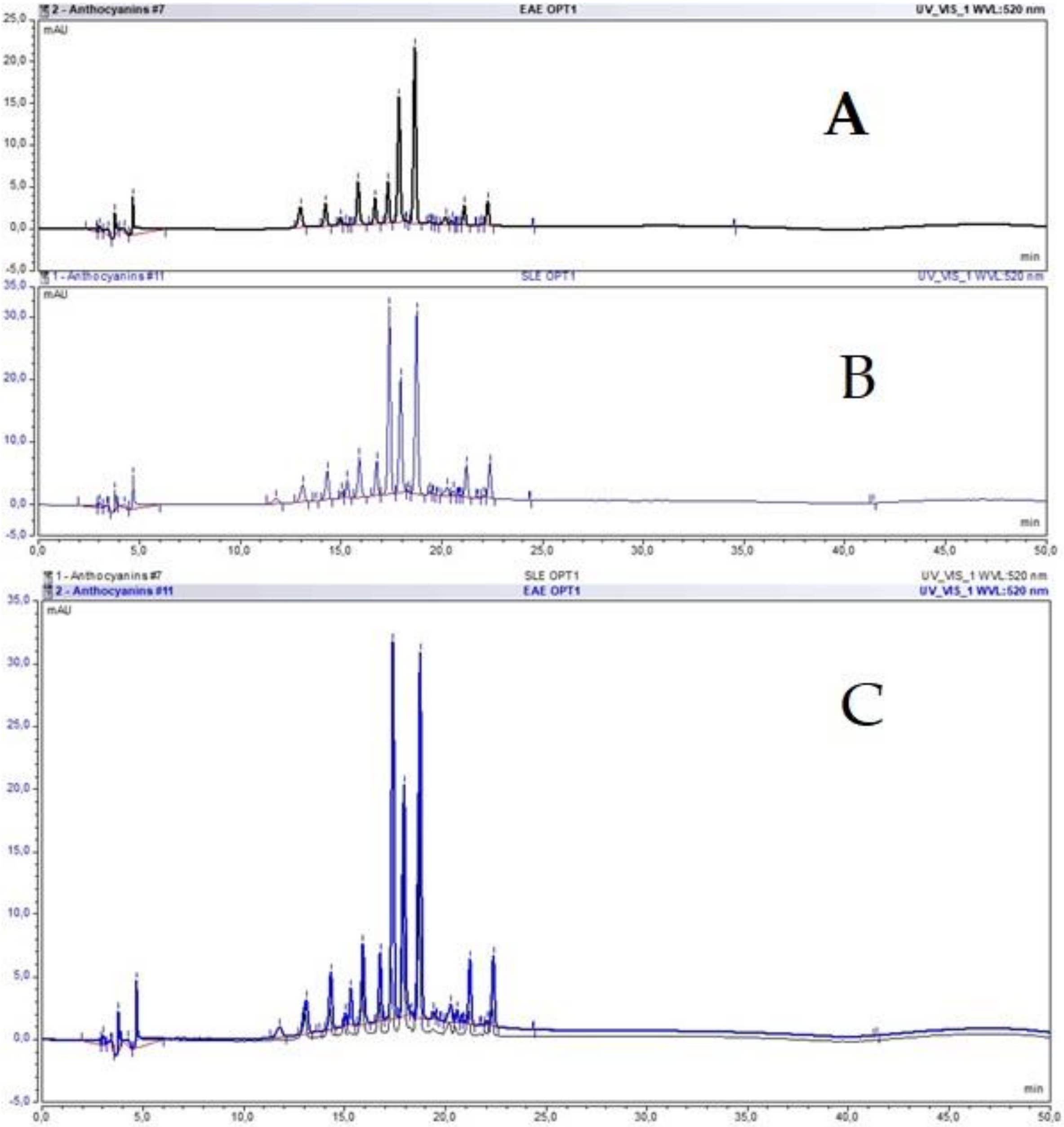

Figure 1. HPLC chromatograms at 520 nm of SLE and EAE extracts under optimal conditions. (**A**) EAE extract (**B**) SLE extract, and (**C**) overlaid chromatograms.
